# Alternations of interhemispheric functional connectivity in patients with acute acquired concomitant esotropia: a resting state fMRI study using voxel-mirrored homotopic connectivity

**DOI:** 10.3389/fnins.2024.1515675

**Published:** 2025-01-06

**Authors:** Jiayu Chen, Jie Hao, Jiawen Liu, Huijian Li, Zhaojun Meng, Jing Fu

**Affiliations:** ^1^Beijing Tongren Eye Center, Beijing Tongren Hospital, Beijing Key Laboratory of Ophthalmology & Visual Sciences, Capital Medical University, Beijing, China; ^2^Wilmer Eye Institute, School of Medicine, Johns Hopkins University, Baltimore, MD, United States

**Keywords:** acute acquired concomitant esotropia (AACE), resting-state functional magnetic resonance imaging (rs-fMRI), voxel-mirrored homotopic connectivity (VMHC), functional connectivity, interhemispheric

## Abstract

**Purpose:**

To investigate the changes in cerebral hemispheric functional connections in patients with acute acquired concomitant esotropia (AACE) and their relationship with clinical manifestations, utilizing voxel-mirrored homotopic connectivity (VMHC).

**Methods:**

A prospective, observational study was conducted involving 32 AACE patients and 31 age-, sex-, and education-matched healthy controls (HC). The resting-state functional magnetic resonance imaging (rs-fMRI) signals, binocular vision function, and psychometric scale scores were collected rs-fMRI data and structural image data were analyzed for VMHC, and a two-sample *t*-test was used to analyze the differences in VMHC between groups. Spearman correlation analysis evaluated the relationship between fMRI indicators and clinical features.

**Results:**

There was no statistical difference between the two groups concerning sex, age, height and weight. VMHC levels in the superior frontal gyrus and anterior cingulate were significantly lower in the AACE group (*p* < 0.05). In the AACE group, the VMHC values of the left caudate positively correlated with near vision work duration (*r* = 0.381, *p* = 0.034), the deviation angles at near (*r* = 0.428, *p* = 0.015) and at distance (*r* = 0.416, *p* = 0.018). The VMHC values in the bilateral olfactory cortex also positively correlated with the near vision work duration (Right: *r* = 0.389, *p* = 0.031; Left: *r* = 0.372, *p* = 0.039) while Beck Depression Inventory (BDI) scores negatively correlated with the VMHC values of the left olfactory cortex (*r* = −0.359, *p* = 0.048).

**Conclusion:**

The dysfunction of the medial frontal gyrus and anterior cingulate gyrus is the underlying neuropathological mechanism of AACE, and these dysfunctions may be related to poor eye habits and the severity of deviation.

## 1 Introduction

Acute acquired concomitant esotropia (AACE) is a subtype of esotropia characterized by diplopia and a sudden onset of esotropia (Clark et al., 1989). AACE predominantly affects adults or older adolescents, presenting with normal eye movements and equal deviation angles in all direction. Recently, the incidence of AACE has increased, especially during and after the COVID-19 lockdown (Vagge et al., 2020; Neena et al., 2022; Mohan et al., 2021).

The pathogenesis of AACE is multifaceted and not fully understood, with hypotheses suggesting links to accommodation and convergence-divergence dysfunctions (Shanker and Nigam, 2015). Myopic overcorrection and presbyopia-related accommodation decline are implicated, with the latter showing higher AC/A ratios (Fresina et al., 2016; Tong et al., 2020). AACE’s occurrence in aphakic eyes indicates that non-accommodative factors (Ruatta and Schiavi, 2020), such as extraocular muscle abnormalities, may play a role. Our research reveals significant changes in the size and volume of dominant eye muscles, possibly as a compensatory response to binocular diplopia, with the LR muscle potentially enlarging to counteract increased convergence (Chen et al., 2024). Additionally, the anterior positioning of the medial rectus muscle in AACE patients may cause over-concentration, disrupting the balance between convergence and divergence and leading to esotropia (Cai et al., 2019).

Conversely, the emergence of AACE may also be linked to complex neural network within the visual system. Advances in functional magnetic resonance imaging (fMRI) enable precise functional localization of the visual cortex, allowing for the assessment of central visual functions in AACE patients without intracranial lesions. Previous studies have identified functional deficits in the visual cortex associated with various types of strabismus (Guo et al., 2022; Yan et al., 2019). These abnormalities in spontaneous brain activity, which can be attributed to visual compensation, can also be considered as one of the causative factors of such disorders. Notably, using the amplitude of low-frequency fluctuation (ALFF) in resting-state fMRI, one study found deficits in the primary visual cortex and dorsal pathways in AACE patients, with alterations in the fusiform gyrus correlating with deviation angles, suggesting a connection between AACE and visual processing centers (Hu et al., 2023).

Synchronization between cerebral hemispheres is crucial for visual experience. Voxel-mirrored homotopic connectivity (VMHC) accurately and efficiently assesses changes in functional connectivity between hemispheres related to a patient’s behavior and cognition by measuring correlations between hemispheric blood oxygen level-dependent time series that reflect the pattern of information exchange and integration between hemispheres (Mancuso et al., 2019). Recent studies have increasingly focused on VMHC, establishing its associations with a variety of diseases and functional states. It has been applied to analyze numerous ocular conditions, including primary open-angle glaucoma (Wang et al., 2018), monocular blindness (Shao et al., 2018), strabismic amblyopia (Peng et al., 2021; Zhang et al., 2021; Liang et al., 2017), high myopia (Cheng et al., 2022), blepharospasm (Wei et al., 2018), unilateral acute open globe injury (Ye et al., 2018), optic neuritis (Song et al., 2023), concomitant exotropia (Zhang et al., 2018), and congenital nystagmus (Wen et al., 2023).

The objective of our study is to utilize VMHC analysis to detect functional brain changes in patients with AACE. We aim to identify whether these alterations in brain functional connection impact visual quality and overall quality of life, thereby offering novel insights into the neural underpinnings of AACE. This approach is pivotal for advancing our understanding of the condition.

## 2 Materials and methods

### 2.1 Participants

The study population consisted of patients diagnosed with AACE at Beijing Tongren Hospital (Beijing, China) from January 2021 to December 2022, along with healthy subjects recruited from the local community. A total of 32 patients with AACE (15 males and 17 females) and 31 HC (10 males and 21 females) were included in this study. This study adhered to the principles outlined in the Declaration of Helsinki and received approval from the Ethics Committee of Beijing Tongren Hospital (TRECKY2021-228), and registered with the China Clinical Trials Registry (ChiCTR2100053717).

The inclusion criteria for both the AACE group and the HC group were as follows: (1) Subjects in both groups exhibited symptoms of diplopia or acute episodes of esotropia and were diagnosed with AACE by an experienced clinician for the AACE group, while the HC group was required to match the AACE patients in terms of gender, age, years of education, handedness, height, and weight; (2) All subjects cooperated with the study examinations; (3) Informed consent was provided by the subjects themselves or through a legal guardian.

The exclusion criteria for both groups were as follows: (1) Presence of developmental abnormalities, cranial or neurological diseases, or a history of head trauma; (2) History of neuropsychiatric disorders or use of psychotropic medications in the past month; (3) History of drug or alcohol addiction or abuse; (4) other organic eye diseases; (5) Inability to cooperate with all required examinations.

### 2.2 Ophthalmic examination

All participants underwent comprehensive ophthalmological examinations, including assessment of best-corrected visual acuity (BCVA), refractive status, fundus examination, synoptophore testing for binocular vision and stereoacuity evaluation, as well as alternate cover test to assess eye alignment. The spherical equivalent (SE) was calculated by summing the sphere power with half of the cylinder power (sphere +0.5 × cylinder). The angles of deviation were measured at near fixation (1/3 m) and distance fixation (6 m) using a prism in combination with alternative cover testing.

Binocular vision at distance fixation, encompassing simultaneous vision, fusion, and distance stereopsis, was evaluated using a synoptophore device (CLEMENT-CLARKE, UK; type 2001), with the normal simultaneous vision range is defined as −3° ~ +3°. Fusion and distance stereopsis were qualitative measured. Near stereopsis was assessed using a Random Dot Stereogram (RDS) at an optimal viewing distance of 40 cm, with patients achieving results within or below 60 s classified as having good near stereopsis.

### 2.3 Questionnaire survey

The age of onset and duration of the AACE in all patients was determined based on self-reported information. Patients were extensively questioned about their diplopia symptoms, which were categorized as follow: distance only, near only, or both distance and near. Additionally, patients were queried as about any history of near vision work prior to the onset of the disease, and if applicable, the daily duration of near vision work was recorded.

All patients completed the Beck Depression Inventory-II (BDI-II) (Wang and Gorenstein, 2013), the Montreal Cognitive Assessment (MoCA) (Kang et al., 2018), and the State–Trait Anxiety Inventory (STAI) (Marteau and Bekker, 1992). The STAI consists of two scales: the State Anxiety Inventory (S-AI) scale, which measures the severity of current anxiety symptoms, and the Trait Anxiety Inventory (T-AI) scale, which assesses the subject’s typical or general anxiety levels.

### 2.4 Resting-state fMRI parameters

All the subjects underwent scanning using a 3-Tesla MRI scanner (MAGNETOM Prisma, Siemens Healthcare, Erlangen, Germany) equipped with a standard 64-channel head coil. Foam pads were strategically placed around the subjects’ heads to minimize any potential head motion artifacts during the scan, while earplugs were provided to attenuate scanner noise.

Three-dimensional T1-weighted anatomical images were acquired using a 3D magnetization-prepared rapid gradient-echo imaging sequence, with the following scan parameters: repetition time = 2000 ms; echo time = 2.25 ms; flip angle = 8°; field of view = 256 mm × 256 mm; in-plane image resolution = 1 mm × 1 mm; slice thickness = 1 mm, no gap; and 192 continuous sagittal slices.

Functional images were obtained with the following parameters: repetition time = 1,000 ms; echo time = 33 ms; flip angle = 64°; field of view = 208 mm × 180 mm; in-plane image resolution = 2 mm × 2 mm; matrix size = 104 × 90; slice thickness = 2 mm; slice gap = 0.4 mm; and 60 slices.

### 2.5 VMHC statistical analysis

Functional data were analyzed with the Data Processing Assistant for Resting-State fMRI Advanced Edition (DPARSFA^1^) and Statistical Parametric Mapping (SPM12)^2^ based on MATLAB R2016a (Mathworks, Natick, MA).

To enhance normality, the individual VMHC maps were transformed into z values using a Fisher z-transformation with REST software^3^. Subsequently, the global VMHC was computed and included as a covariate in the subsequent statistical analysis. The Anatomical Automatic Labeling (AAL) 3 version 1 was used to extract the average value of the quantitative indicators of all brain regions, and the graph was drawn using the XjView toolbox.

### 2.6 Statistical analysis

The difference in the z-maps VMHC between the AACE groups and the health controls was examined with two-sample t-tests in the SPM8 toolkit (*p* < 0.001 for multiple comparisons using Gaussian Random Field theory). SPSS software (version 27.0; IBM, IL, United States). The chi-square test was used for categorical variable differences (*p* < 0.05), and two-sample independent test or non-parametric test was used for continuous variable differences (*p* < 0.05). Spearman’s correlation analysis evaluated the relationship between fMRI indicators and clinical features in abnormal areas (*p* < 0.05).

## 3 Results

### 3.1 Demographics and visual measurements

No significant differences were observed between the two groups regarding gender, age, height, and weight (Table 1). Patients in the AACE group had a mean age of onset of 33.28 ± 9.15 years and a mean disease duration of 2.99 ± 2.26 years. Their mean angle of deviation was 29.22 ± 20.44Δ at near and 29.78 ± 20.44Δ at distance. The AACE group had worse binocular visual function than the normal group, but there was no significant difference in spherical equivalent and time of near vision work (Table 1). The psychological scale scores for both groups are presented in Table 2.

**Table 1:** Demographic and clinical characteristics of patients in AACE and HC groups.

		AACE (*N* = 32)	HC (*N* = 31)	*t/*Chi2	*P*
		Mean (SD)/*n* (%)	Mean (SD)/*n* (%)		
Sex	Male	15 (46.9)	10 (32.3)	1.406^b^	0.236
	Female	17 (53.1)	21 (67.7)		
Age/year		33.28 (9.15)	33.45 (10.83)	−0.068^a^	0.946
Height/cm		167.48 (9.56)	166.43 (7.80)	0.472^a^	0.638
Weight/kg		64.48 (13.94)	61.40 (15.37)	0.829^a^	0.411
Duration of Education/year		15.31 (2.38)	16.55 (3.29)	−1.712^a^	0.092
SE-R		−5.13 (2.70)	−4.14 (3.16)	−1.145^a^	0.258
SE-L		−5.09 (2.56)	−3.77 (3.33)	−1.538^a^	0.131
Simultaneous vision (°)		17.94 (7.27)	0.25 (1.67)	12.342^a^	<0.001**
Collective fusion (°)		8.21 (7.29)	−4.88 (10.36)	4.095^a^	<0.001**
Diffuse fusion (°)		28.86 (6.77)	13.5 (9.20)	5.258^a^	<0.001**
Fusion range (°)		19.97 (8.52)	25.38 (4.66)	−1.717^a^	0.095
Distance stereopsis	+	17	31	19.072^b^	<0.001**
	−	15	0		
Near stereopsis	+	11	31	30.516^b^	<0.001**
	−	21	0		
Time of near vision work (hours/day)		8.15 (2.54)	7.05 (3.40)	1.585^a^	0.119
Age of onset/year		29.66 (9.65)	N/A	N/A	N/A
Duration of disease/year		2.99 (2.26)	N/A	N/A	N/A
The angles of deviation at near/Δ		29.22 (20.44)	N/A	N/A	N/A
The angles of deviation at distance/Δ		29.78 (20.44)	N/A	N/A	N/A

Data are presented as mean ± SD and *N*/%; AACE, acute acquired concomitant esotropia. N/A, not applicable. ^a^Indicates independent samples *t*-test; ^b^indicates Chi-square test. *Statistically subnormal (*p* < 0.05). **Statistically subnormal (*p* < 0.001); R, right; L, left.

**Table 2:** Scores of physical scales of patients in AACE and HC groups.

Scales Mean (SD)	AACE (*N* = 32)	HC (*N* = 31)	*t*	*P*
BDI-II	5.42 (6.91)	6.11 (3.48)	−0.402	0.690
MoCA	27.33 (1.17)	28.67 (1.54)	−3.064	0.004*
STAI				
S-AI	44.33 (5.23)	46.71 (3.94)	−1.843	0.071
T-AI	42.07 (5.18)	45.75 (5.07)	−2.622	0.011*

*Statistically subnormal (*P* < 0.05).

### 3.2 VMHC differences

**Figure 1:**
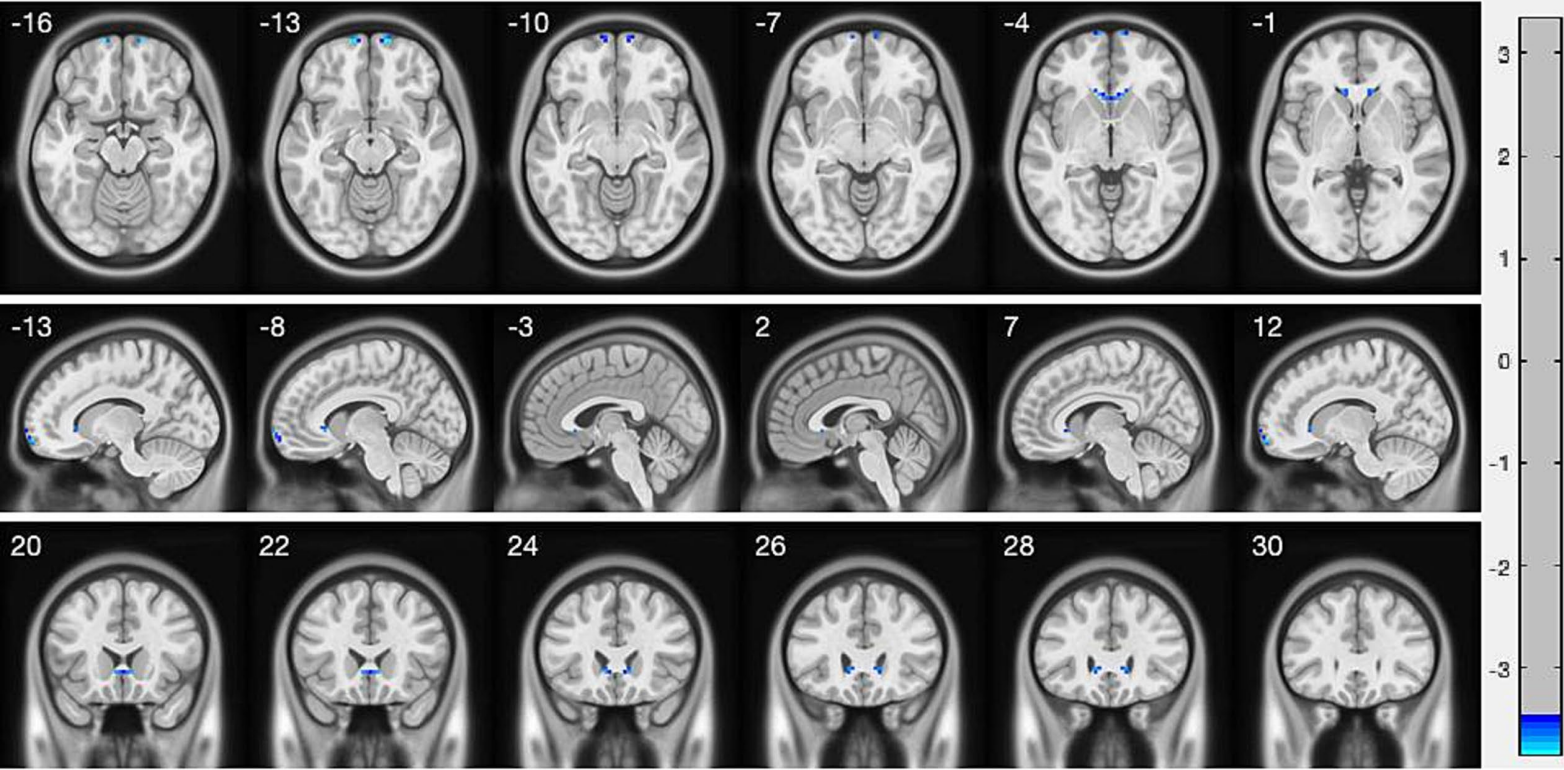
Interhemispheric connectivity in the CSA versus NCs. Blue areas indicated the lower VMHC values showed *p* < 0.001.

**Figure 2:**
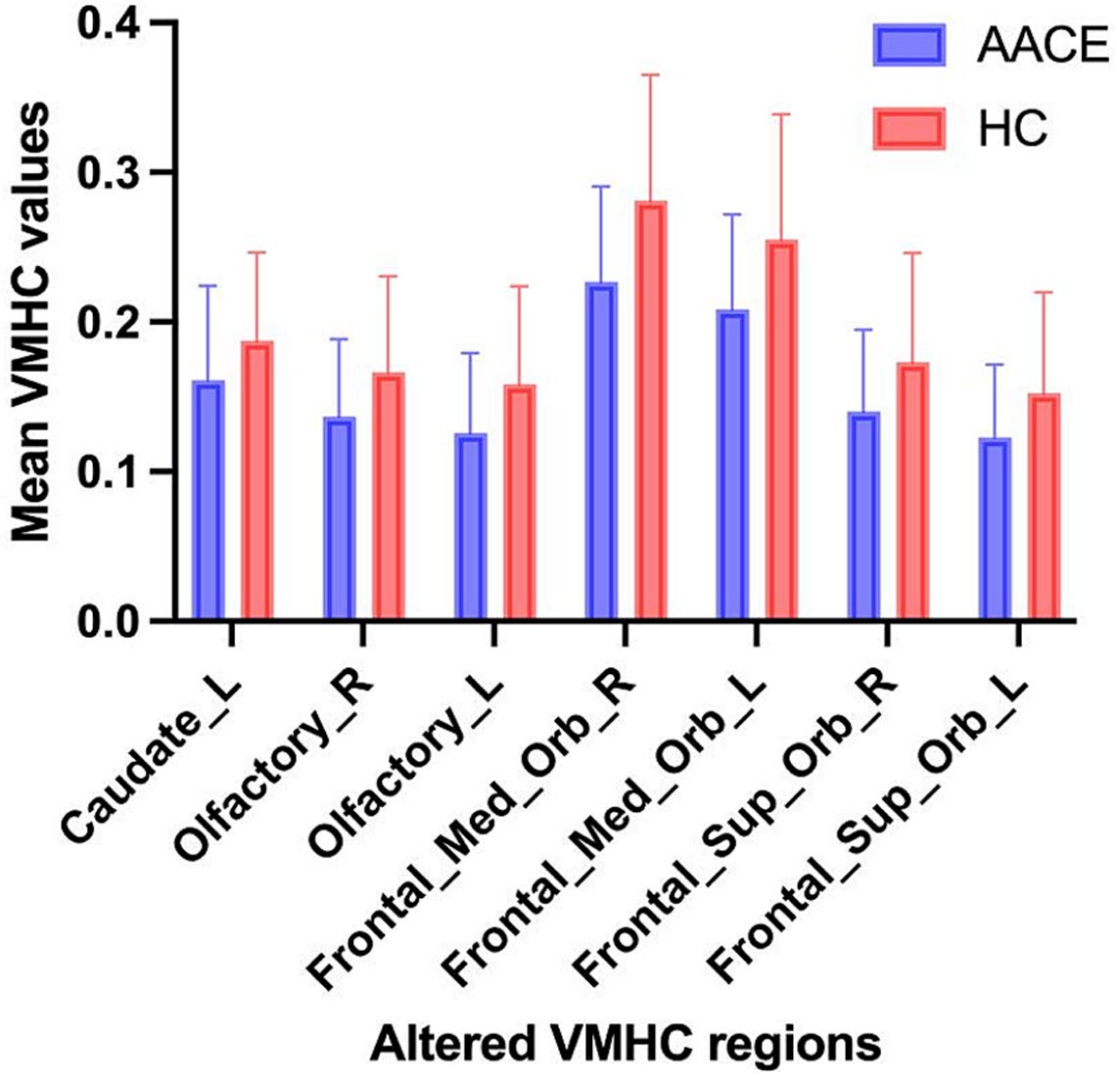
The mean values of altered VMHC values between the AACE and HC groups. Data presented as mean ± standard deviation.

Compared with HC, AACE patients had significantly lower VMHC values in the superior frontal gyrus (SFG) and anterior cingulate gyrus (ACG) (Figure 1 and Table 3). Differences between the two groups appeared in Brodmann area 11 and Brodmann area 24. Figure 2 shows the significantly different VMHC values between the two groups.

**Table 3:** Brain regions with significant changes in regional fMRI (VMHC) between patients with AACE and HC groups.

Measurements	Brain regions	Brodmann area	Peak MNI coordinate			Cluster size (voxel)	Peak *t*-value
			*x*	*y*	*z*		
VMHC	Frontal_Med_Orb_R/Frontal_Sup_Orb_R	11	15	66	−12	14	−3.8679
	Frontal_Sup_Orb_L/Frontal_Med_Orb_L	11	−15	66	−12	12	−3.8679
	Olfactory_R/Olfactory_L/Caudate_L	24	9	24	−3	17	−3.7192

The statistical threshold is set at the voxel level, and the GRF theory is used for multiple comparisons (cluster-level of *P* < 0.001 and cluster ≥ 10 voxels).

### 3.3 Correlation analysis

**Figure 3:**
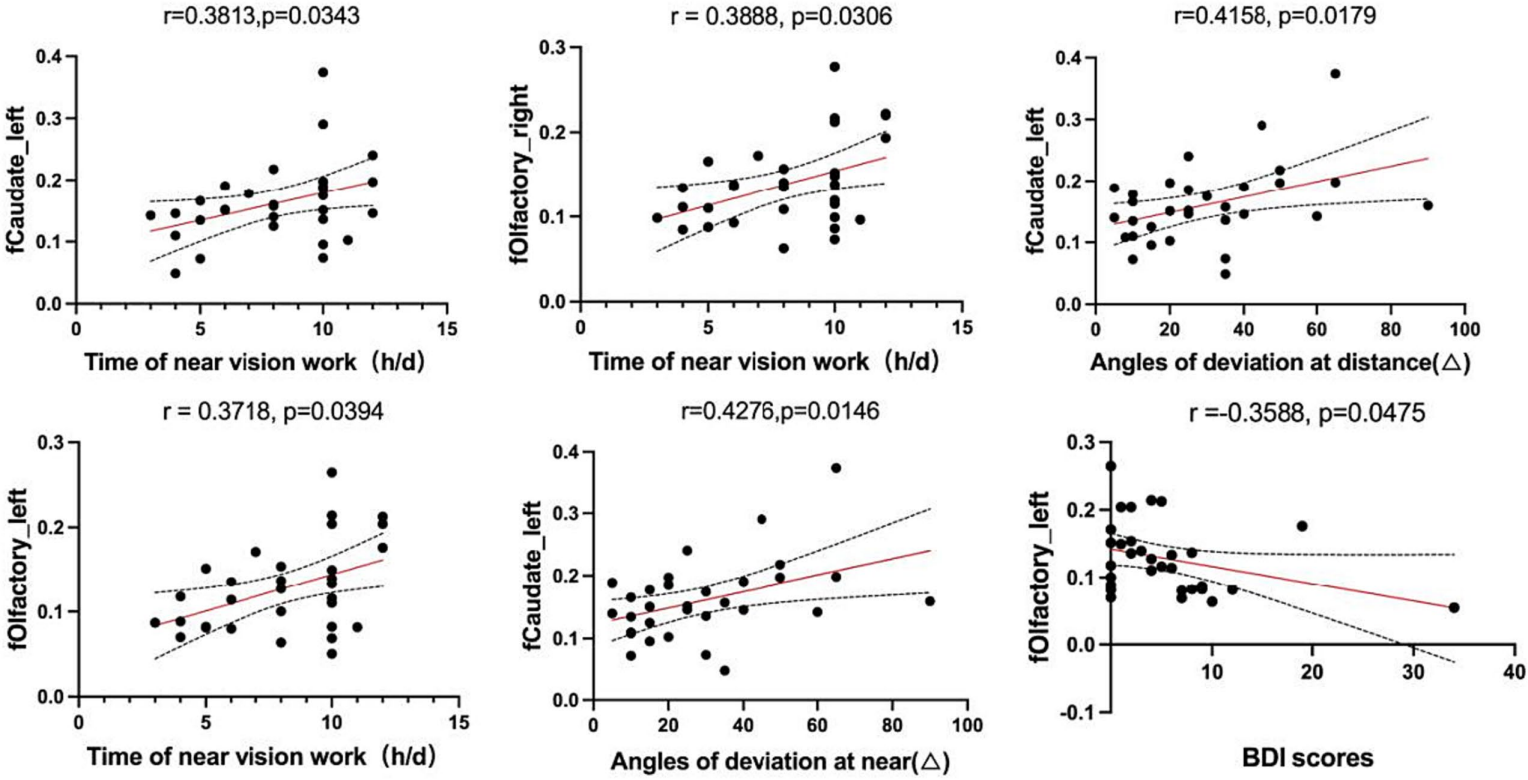
Correlations between the values of fMRI in abnormal brain regions and clinical features.

In the AACE group, the VMHC values of the left caudate positively correlated with the time of near vision work (*r* = 0.3813, *p* = 0.0343), the angles of deviation at near (*r* = 0.4276, *p* = 0.0146) and at distance (*r* = 0.4158, *p* = 0.0179). The VMHC values of the bilateral olfactory cortex positively correlated with the duration of near vision work (Right: *r* = 0.3888, *p* = 0.0306; Left: *r* = 0.3718, *p* = 0.0394) while the BDI scores showed a negative correlation with the VMHC values of the left olfactory cortex (*r* = −0.3588, *p* = 0.0475) (Figure 3).

## 4 Discussion

This study first utilized the VMHC method to survey functional network brain activity changes in AACE patients. We observed reduced VMHC values in the SFG and ACG in patients with AACE compared to HC, and these reduced values correlated with time of near vision work, angles of deviation at near and distance and BDI scores.

The frontal lobe is located at the front of the cerebral hemispheres and is the most evolved part of the developing brain (Neulinger et al., 2016). Damage to this area can lead to impairments in voluntary movement, language expression and memory. Among these, supplementary eye field (SEF), located in the medial frontal cortex, is involved in the control of random eye movements and are particularly associated with eye tracking (Purcell et al., 2012). Activity in the frontal eye fields (FEF) was also observed during visual search (Lowe et al., 2022). The superior frontal gyrus (SFG) is located superiorly in the prefrontal cortex and is thought to consist of several cytoarchitecturally distinct subregions, including Brodmann’s zones 6, 8, 9, and 32 (Petrides and Pandya, 1999; Petrides and Pandya, 2002). Functional impairment was found in the above areas in all types of strabismus (Huang et al., 2016a; Tan et al., 2016; Tan et al., 2018; Huang et al., 2018).

One study found significantly higher levels of VMHC in some brain regions in the middle frontal lobe of patients with concomitant exotropia, possibly reflecting abnormal activity in the kinetic eye area due to a compensatory mechanism that may not be the cause of strabismus, but rather a consequence of strabismus (Yan et al., 2019). Besides, another study found that patients with strabismus and amblyopia had significantly lower VMHC values in the bilateral frontal super orbits and bilateral frontal gyrus, which is similar to our findings (Peng et al., 2021). A previous study from our team revealed fusional vergence dysfunction is present in adult AACE cases (Zhao et al., 2022). All of the patients in this study had varying degrees of diplopia symptoms that did not resolve on their own after rest. They had significant abnormalities in fusion function. Since it has been found that the bilateral frontal gyrus regulates normal fusion function in human eyes (Xubo et al., 2014), we hypothesized that a decrease in frontal VMHC, resulting in fusion dysfunction, contributes to the development of AACE. This may be the difference between AACE and other types of strabismus, especially concomitant exotropia.

The cingulate gyrus, situated within the limbic system, has been linked to the formation of mood, the experience of depression and the perception of pain (Ebert and Ebmeier, 1996). The limbic system is intimately associated with memory and emotion. The anterior cingulate gyrus plays a role in a number of established functions, including emotion, cognition, locomotion, visceral movement, maternal behavior and social interaction (Apps et al., 2016; Bliss et al., 2016; Zou et al., 2021). A study utilizing the DTI technique to investigate brain-wide microstructural alterations in individuals with common strabismus revealed that patients with this condition exhibited markedly elevated mean diffusivity values in the left anterior cingulate gyrus, indicating microstructural modifications in this region (Huang et al., 2016a). Patients with common strabismus were also observed to have higher regional homogeneity (ReHo) values were observed in the bilateral cingulate gyrus in the study by Huang et al. (2016b). Additionally, some researchers have discovered that the diffusion coefficient (DC) values in the anterior cingulate cortex (ACC) were markedly elevated in adult patients with common external strabismus in comparison to controls, indicating that it may be a contributing factor to anterior cingulate gyrus dysfunction (Tan et al., 2018). Meanwhile, some researchers have found that optic neuritis patients had reduced VMHC values in the left middle cingulate gyrus, suggesting that functional abnormalities in the cingulate gyrus may lead to cognitive decline or loss in patients (Song et al., 2023).

Numerous studies have found increased signal levels of various indicators in the cingulate gyrus in patients with exotropia, abnormal activation of the cingulate gyrus to compensate for the impairment of fusional function due to exotropia. One study found higher bilateral cingulate VMHC values in strabismic amblyopia than in the HC group, due to a compensatory increase in visual input deficits caused by strabismic amblyopia (Zhang et al., 2021). However, a study of functional brain connectivity in patients with exotropia found no changes in the cingulate gyrus. In the present study, the VMHC values of the cingulate gyrus in AACE patients were significantly lower than those of the normal group, suggesting that our dysfunctional functional connectivity between the bilateral cingulate gyrus may be an important factor affecting the control of the direction and eye position of strabismus.

In our study, the VMHC signal values in the anterior cingulate gyrus were significantly lower in the AACE group compared to the control group and exhibited a negative correlation with BDI depression scores. Prior research has indicated a correlation between olfactory bulb volume and depression (Sabiniewicz et al., 2022). Concurrently, numerous studies have indicated that individuals with strabismus frequently exhibit psychological irregularities, including depressive symptoms (Lin et al., 2014; Lee et al., 2022). Consequently, we postulate that strabismus may be linked to anterior cingulate gyrus dysfunction, which could elucidate the prevalence of depressive symptoms in patients with strabismus. This underscores the necessity for greater emphasis on the psychological well-being and quality of life of patients, in addition to conventional diagnosis and treatment in the clinic.

It is noteworthy that the VMHC values of the left caudate in the AACE group exhibited a positive correlation with the duration of near vision work, the angles of deviation at near and at distance. Additionally, the VMHC values of the bilateral olfactory cortex demonstrated a positive correlation with the duration of near vision work. Excessive near vision work may be a significant contributing factor to the development of AACE (Topcu Yilmaz et al., 2020). It is possible that excessive tension in the medial rectus muscle, caused by near vision work, may result in esotropia if the fusion force is insufficient to overcome this tension. Alternatively, prolonged use of smartphones may stimulate the ciliary muscle, leading to convergence spasms and the development of AACE (Kaur et al., 2019).

Although the present study revealed functional connectivity differences between AACE patients and healthy controls in the resting state by means of fMRI, it remains unclear what association exists between this and the visual impairment associated with strabismus. A more in-depth longitudinal study of brain changes after strabismus correction will be conducted in future investigations.

The VMHC values of the frontal and marginal lobes of AACE patients were changed, suggesting that dysfunction of the medial frontal gyrus and anterior cingulate gyrus may lead to fusion dysfunction, thereby contributing to the development of AACE. This impairment this is related to poor eye habits and the severity of strabismus.

## Data Availability

The raw data supporting the conclusions of this article will be made available by the authors, without undue reservation.

## Footnotes

^1^ http://rfmri.org/DPARSFA

^2^ https://www.fil.ion.ucl.ac.uk/spm/

^3^ http://resting-fmri.sourceforge.net
